# Distinct Microbial Community Performing Dissimilatory Nitrate Reduction to Ammonium (DNRA) in a High C/NO_3_^−^ Reactor

**DOI:** 10.1264/jsme2.ME17193

**Published:** 2018-09-29

**Authors:** Pokchat Chutivisut, Kazuo Isobe, Sorawit Powtongsook, Wiboonluk Pungrasmi, Futoshi Kurisu

**Affiliations:** 1 Department of Environmental Engineering, Faculty of Engineering, Chulalongkorn University Bangkok Thailand; 2 Department of Applied Biological Chemistry, Graduate School of Agricultural and Life Sciences, The University of Tokyo Tokyo Japan; 3 Center of Excellence for Marine Biotechnology, Department of Marine Science, Faculty of Science, Chulalongkorn University Bangkok Thailand; 4 National Center for Genetic Engineering and Biotechnology, National Science and Technology Development Agency Pathum Thani Thailand; 5 Research Center for Water Environment Technology, Graduate School of Engineering, The University of Tokyo Tokyo Japan

**Keywords:** dissimilatory nitrate reduction to ammonium (DNRA), semi-continuous sequencing batch reactor, C/NO_3_^−^ ratio, stable-isotope tracer, Illumina MiSeq 16S rRNA sequencing

## Abstract

A dissimilatory nitrate reduction to ammonium (DNRA) microbial community was developed under a high organic carbon to nitrate (C/NO_3_^−^) ratio in an anoxic semi-continuous sequencing batch reactor (SBR) fed with glucose as the source of carbon and NO_3_^−^ as the electron acceptor. Activated sludge collected from a municipal wastewater treatment plant with good denitrification efficiency was used as the inoculum to start the system. The aim of this study was to examine the microbial populations in a high C/NO_3_^−^ ecosystem for potential DNRA microorganisms, which are the microbial group with the ability to reduce NO_3_^−^ to ammonium (NH_4_^+^). A low C/NO_3_^−^ reactor was operated in parallel for direct comparisons of the microbial communities that developed under different C/NO_3_^−^ values. The occurrence of DNRA in the high C/NO_3_^−^ SBR was evidenced by stable isotope-labeled nitrate and nitrite (^15^NO_3_^−^ and ^15^NO_2_^−^), which proved the formation of NH_4_^+^ from dissimilatory NO_3_^−^/NO_2_^−^ reduction, in which both nitrogen oxides induced DNRA activity in a similar manner. An analysis of sludge samples with Illumina MiSeq 16S rRNA sequencing showed that the predominant microorganisms in the high C/NO_3_^−^ SBR were related to *Sulfurospirillum* and the family *Lachnospiraceae*, which were barely present in the low C/NO_3_^−^ system. A comparison of the populations and activities of the two reactors indicated that these major taxa play important roles as DNRA microorganisms under the high C/NO_3_^−^ condition. Additionally, a beta-diversity analysis revealed distinct microbial compositions between the low and high C/NO_3_^−^ SBRs, which reflected the activities observed in the two systems.

Dissimilatory nitrate (NO_3_^−^) reduction is part of the nitrogen cycle driven by microorganisms with the ability to use NO_3_^−^ as their electron acceptor. Known dissimilatory NO_3_^−^-reducing pathways include denitrification, which generates nitrogenous gases as its products, and dissimilatory nitrate reduction to ammonium (DNRA), which produces ammonium (NH_4_^+^). Both of these processes occur under anoxic environments and utilize the same types of electron donors, such as organic carbon, sulfur, and iron (6, 12, 28). Therefore, denitrifiers and DNRA microorganisms are competitors for electron donors and electron acceptors (NO_3_^−^ and nitrite [NO_2_^−^]), as well as for the habitats in which they grow. Although the microorganisms responsible for denitrification have been extensively examined, limited information is currently available on the DNRA microbial group.

Although different nomenclatures have been termed for this dissimilatory NH_4_^+^-forming process, including NO_3_^−^ or NO_2_^−^ ammonification, it is mainly known as DNRA. This microbial group may be further classified into respiratory and fermentative types (7, 22), which use different metabolic pathways to catalyze the formation of NH_4_^+^. DNRA microorganisms have been hypothesized and observed to occur in a high electron donor, limited NO_3_^−^ environment, which is normally referred to as a high carbon to NO_3_^−^ (C/NO_3_^−^) condition, whereas denitrifiers appear to prefer a low C/NO_3_^−^ condition (7, 34). Thermodynamically, the energy gained per mole electron donor and per mole NO_3_^−^ differs for denitrification and DNRA, with the former obtaining a higher amount of energy per glucose molecule and the latter gaining more energy per NO_3_^−^ molecule (32). These findings suggest how DNRA microorganisms compete with denitrifiers in a NO_3_^−^- limiting ecosystem. The influence of the C/NO_3_^−^ ratio on NO_3_^−^-reducing communities or a pure culture was recently reported (23, 36–38, 44), with selective pressure of the ratio on the NO_3_^−^ reduction pathways being observed.

In agriculture, DNRA is considered to be a beneficial process because it preserves nitrogen fertilizers within soil (29). However, this pathway is undesirable in a biological wastewater treatment system due to its production of NH_4_^+^, a waste product that is generally removed by nitrification-denitrification. The growth and activity of DNRA microorganisms decrease denitrification efficiency, and increase the waste load on nitrification as well as total nitrogen discharged with the effluent. DNRA activity was previously detected in anaerobic digester systems (1, 2, 13, 21), a pilot plant treating sulfate (SO_4_^2−^)- and NO_3_^−^-containing wastewater (14), a lab-scale denitrifying reactor (4), and aquaculture nitrogen removal systems (10). The extent of DNRA measured among these studies suggests its competitive potential in high organic-loaded wastewaters; however, the microorganisms responsible for the process have rarely been identified in these environments.

In order to control the occurrence of microorganisms with the capacity for DNRA within a wastewater treatment system, it is essential to recognize their identity and diversity as the first step towards maneuvering these populations to the required process. Therefore, in order to identify potential DNRA microorganisms in wastewater ecosystems, an anoxic semi-continuous sequencing batch reactor (SBR) maintained at a high C/NO_3_^−^ ratio was applied to monitor subsequent microbial community adaptation when using activated sludge as the inoculum. In a direct comparison, a low C/NO_3_^−^ reactor was operated under otherwise the same environmental conditions and using the same inoculum. The objectives of the present study were (i) to investigate the presence and activity of the DNRA pathway under low and high C/NO_3_^−^ anoxic sludge ecosystems and (ii) to identify the composition of the resulting microbial communities using an Illumina MiSeq 16S rRNA sequencing analysis. In order to trace the occurrence of DNRA, the stable-isotope tracers ^15^NO_3_^−^ and ^15^NO_2_^−^ were utilized to track the formation of ^15^NH_4_^+^ in low and high C/NO_3_^−^ SBR sludge samples.

## Materials and Methods

### Operation of the reactors

The two reactors were maintained under low and high C/NO_3_^−^ ratios, respectively, and operated in the semi-continuous sequencing batch mode, which provided environments for the growth of microorganisms favored under each condition. During each cycle, the systems were set to the continuous feeding batch mode in order to maintain stable concentrations of substrates in the reactors, while also retaining a large amount of biomass sludge in each SBR. Both SBRs were started with an inoculum (1,393±42 mg mix liquor suspended solids [MLSS] L^−1^) from a municipal wastewater treatment plant operated under an anaerobic/oxic/anoxic/oxic (AOAO) process exhibiting good denitrification efficiency with a nitrogen removal rate of approximately 60%. SBRs were run in 1-L working volume reactors with six-blade turbine stirrers, and were maintained at 20°C in a control temperature room. Mixing was performed at 150 rpm in order to maintain a homogenous state inside the systems. During days 1–15 of the operation, the reactor cycle was set to 6 h, which comprised 320 min of continuous feeding and mixing, 30 min of sludge settling, and 10 min of effluent withdrawal. Only unsettled sludge was removed with the discharged effluent during the end of the cycle. The volume exchanged per cycle was set at 1/4 of the reactor working volume, whereas the flow rate was set at 47 mL h^−1^ (equal to a dilution rate of 0.047 h^−1^), resulting in a hydraulic retention time (HRT) of 24 h. After day 15, the cycle was changed to 12 h (680 min of continuous feeding and mixing) while the exchanged volume remained the same; therefore, the flow rate was adjusted to 22 mL h^−1^ during this period (equal to a dilution rate of 0.022 h^−1^) with 48 h of HRT. The flow rates of the influent and effluent were controlled by peristaltic pumps (Masterflex; Cole-Parmer, Chicago, IL, USA), whereas the reactor cycle was set using timers. The solid retention times (SRT) of each reactor during the operation were 3.40±0.45 and 2.52±0.97 d for the low and high C/NO_3_^−^ systems, respectively.

Glucose was selected as the carbon and energy source for microbial growth, and its concentration was varied in order to achieve COD/NO_3_^−^-N ratios of 4/1 and 8/1 for the low and high C/NO_3_^−^ SBRs, respectively (for details, see Table S1). NO_3_^−^ was supplied at the same concentration in both systems. The medium fed to the reactors during the 6-h cycle period was composed of the following (L^−1^): 90 mg of MgSO_4_·7H_2_O, 160 mg of MgCl_2_·6H_2_O, 42 mg of CaCl_2_·2H_2_O, 122 mg of peptone, 20 mg of yeast extract, 50 mg of NH_4_Cl, 11.33 mg of KH_2_PO_4_, 25.67 mg of Na_2_HPO_4_·12H_2_O (15), and 0.3 mL of nutrient solution (40). The concentrations of all nutrients were doubled during the 12-h cycle in order to obtain the same loading as previously. Media were autoclaved and later supplemented with filter-sterilized glucose solution in order to meet the specified COD/NO_3_^−^-N ratio for each reactor. Media were then flushed with argon (Ar) gas for 30–60 min (depending on the volume prepared) before being added to the system.

### Chemical analysis

Water samples were collected daily from both SBRs in order to monitor changes in inorganic nitrogen and organic carbon in the systems. Samples were initially filtered through a glass microfiber filter with a pore size of 0.7 μm (Grade GF/F; GE Healthcare, Little Chalfont, UK) before the chemical analysis. The concentrations of NO_3_^−^, NO_2_^−^, and SO_4_^2−^ were analyzed by ion chromatography (IC) equipped with an anion column (861 Advanced Compact IC; Metrohm, Herisau, Switzerland), whereas NH_4_^+^ was measured by IC with a cation column (761 Advanced Compact IC; Metrohm). Organic carbon was monitored in the form of dissolved organic carbon (DOC) using a total carbon analyzer (TOC-V; Shimadzu, Kyoto, Japan). Changes in biomass were quantified in terms of MLSS as described by the Standard Methods for the Examination of Water and Wastewater (3). The pH of each withdrawn sample was measured using a pH meter.

### Stable isotope incubations

The stable isotope tracers, ^15^NO_3_^−^ and ^15^NO_2_^−^, were applied to verify the occurrence and extent of DNRA in the low and high C/NO_3_^−^ SBR samples. This was performed by monitoring the amount of ^15^NH_4_^+^ produced from the dissimilatory ^15^NO_3_^−^ or ^15^NO_2_^−^ reduction. Sludge for the incubations was taken from the reactors at the end of the operation (on days 60 and 54 for the low and high C/NO_3_^−^ systems, respectively). Experiments were conducted in the batch mode and samples were collected in time series. ^15^NO_3_^−^ and ^15^NO_2_^−^ were used in the form of Na^15^NO_3_ and Na^15^NO_2_ (NLM-157-PK and NLM- 658-PK, respectively, both with 98%+ ^15^N atom; Cambridge Isotope Laboratories). Before the incubations, sludge taken from the reactors was centrifuged to remove the original medium, washed once with fresh medium (same composition as that prepared for SBRs, excluding NH_4_Cl), and then placed into 100-mL serum bottles along with new medium. Serum bottles were closed with butyl rubber stoppers and sealed with aluminum caps before flushing with Ar gas for 10 min. The incubations were then started by injecting either ^15^NO_3_^−^ or ^15^NO_2_^−^ into the serum bottles to a final concentration of 20 mg N L^−1^, along with ^14^NH_4_Cl to a final concentration of 100 mg N L^−1^ and glucose as appropriate to make the same COD/NO_3_^−^-N ratio as in the reactors. All stock solutions were flushed with Ar gas before use. Samples were placed on a rotary shaker set at 150 rpm and temperature was maintained at 20°C. All incubations were performed in triplicate for the sludge of each reactor. The measurement of ^15^NH_4_^+^ produced from the dissimilatory ^15^NO_3_^−^ or ^15^NO_2_^−^ reduction was conducted as previously described (19). Since the original medium from the reactor had been washed off the sludge before the incubations, the amounts of ^14^NO_3_^−^ and ^14^NO_2_^−^ in the samples were considered to be negligible and changes in ^15^NO_3_^−^ and ^15^NO_2_^−^ were measured using the IC method (861 Advanced Compact IC; Metrohm).

### Illumina MiSeq 16S rRNA sequencing analysis of microbial communities

The microbial communities of the low and high C/NO_3_^−^ SBRs were examined using the Illumina MiSeq 16S rRNA sequencing method. Samples analyzed included sludge collected on days 27, 30, 38, 42, and 48 from both reactors as well as the inoculum used to start the systems. Total microbial DNA was extracted from samples using the FastDNA^TM^ SPIN Kit for Soil (MP Biomedicals, Santa Ana, CA, USA) as described by the manufacturer’s protocol. Extracted DNA was then used as a template for PCR amplification with the 341F and 805R primer pair (targeting the V3 and V4 regions of the 16S rRNA gene). The PCR reaction mixture (25 μL) was prepared from a *TaKaRa Ex Taq*^TM^ kit (Takara, Otsu, Japan) with 12 ng of the DNA template, 0.2 μM of each dNTP, 2 mM MgCl_2_, 0.2 μM of each primer, and 1.25 U of *TaKaRa Ex Taq*^TM^. The PCR thermal steps were as follows: 94°C for 3 min; 25 cycles of 94°C for 30 s, 55°C for 30 s, and 72°C for 30 s, and then a final 72°C for 5 min. Four PCR reactions were conducted per sample to reduce bias during PCR amplification. PCR products were then checked by 1.5% (w/v) agarose gel electrophoresis in order to ensure that the correct-sized products were amplified (approximately 460 bp).

Four replicates of each sample were subsequently combined and purified with a NucleoSpin^®^ Gel and PCR Clean-up kit (Macherey- Nagel, Bethlehem, PA, USA) according to the manufacturer’s protocol. The concentrations of the PCR products were examined with a spectrophotometer (NanoDrop^TM^ 2000c Spectrophotometer; Thermo Fisher Scientific, Waltham, MA, USA). The 16S rRNA amplicons obtained were then prepared in a 50-μL final volume reaction containing 5 μL of each Illumina Nextera XT index primer (Illumina, San Diego, CA, USA) and 2X KAPA HotStart ReadyMix (Kapa Biosystems, Wilmington, MA, USA) to index each sample. The PCR thermal steps used were as follows: 94°C for 3 min, followed by 8–10 cycles of 98°C for 20 s, 55°C for 30 s, and 72°C for 30 s, and then a final 72°C for 5 min. The indexed PCR amplicons were then purified with Agencourt AMPure XP beads (Agencourt Bioscience, La Jolla, CA, USA), pooled, and diluted to a final loading concentration of 4 pM. DNA paired-end sequencing was conducted on the Illumina MiSeq platform with MiSeq Reagent Kits v3 (600 cycles; Illumina) at the Omics Sciences and Bioinformatics Center (Chulalongkorn University, Bangkok, Thailand). Sequences were assembled by merging forward and reverse reads, and the operational taxonomic unit (OTU) picking process was performed using QIIME software (version 1.9.0) with an open reference method. Taxonomic assignment from the OTUs to related taxa was performed using UCLUST against the Greengenes database (version 13_8, with a 97% sequence identity threshold). Sequence reads in this study may be accessed at the NCBI Short Read Archive (SRA) under accession numbers SRX3440460–SRX3440470. OTUs with ≥1% and ≥2% maximum relative abundance were selected as representatives for class-level and genus-level analyses, respectively. The beta-diversity of each sample was evaluated in QIIME, and the results obtained were used for the principal coordinate analysis (PCoA) using the weighted UniFrac distance metric (24). Statistical analyses of the results obtained were performed using a two-sample *t*-test on SPSS Statistic ver. 22 (IBM, Armonk, NY, USA).

## Results and Discussion

### Competition between denitrification and DNRA in low and high C/NO_3_^−^ SBRs

In the operation of the reactors, semi-continuous SBRs enriched with NO_3_^−^-reducing communities exhibited the ability to maintain a stable C/NO_3_^−^ ratio during the feeding period (see Fig. S1) as well as the biomass within the systems. Apart from the C/NO_3_^−^ ratio, the applied dilution rate is another factor known to affect competition for NO_3_^−^ in a reactor setting, in which DNRA microorganisms appear to require a low dilution rate for growth (27). This feature may be related to the kinetic aspect of these microorganisms because the requirement for a low dilution rate indicates a long generation time for growth (23), the need for a low NO_3_^−^ concentration to kinetically compete with denitrifiers, or both. Therefore, the dilution rate used in the present study was selected from a range that was expected to allow DNRA to occur. All other operating parameters, besides the glucose concentration, were the same in the low and high C/NO_3_^−^ SBRs in order to allow the C/NO_3_^−^ ratio to be the determining factor for the successful NO_3_^−^-reducing pathway in each ecosystem.

**Fig. 1:**
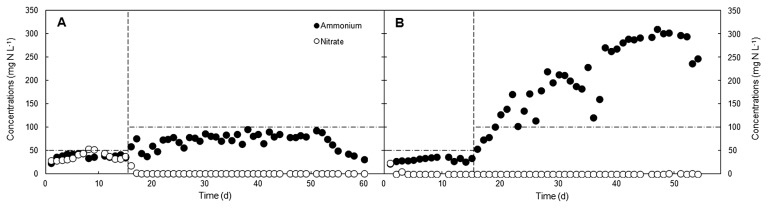
Changes in NO_3_^−^ and NH_4_^+^ concentrations in (A) low and (B) high C/NO_3_^−^ SBRs. Vertical lines indicate the point of change in the SBR cycle, horizontal lines indicate the level of NH_4_^+^ supplied to the reactors.

Competition between the two NO_3_^−^ reduction pathways was initially verified by observing changes in the inorganic nitrogen profiles of the low and high C/NO_3_^−^ SBRs (Fig. 1). During the initial phase of the low C/NO_3_^−^ reactor operation, NO_3_^−^ was detected in the system in the range of 17.7–52.5 mg N L^−1^ (47.8–82.3% NO_3_^−^ conversion) and was consumed to less than 1.0 mg N L^−1^ (>99% NO_3_^−^ conversion) after day 17, which suggested that NO_3_^−^ had become limited for heterotrophic growth or that it was used by autotrophic NO_3_^−^ reducers. NO_2_^−^ was only detected on day 4 (1.7 mg N L^−1^) and then remained below the detection limit (see Fig. S2). The level of NH_4_^+^ (23.2–95.1 mg N L^−1^) was always lower than the added NH_4_^+^ concentration, and, thus, there was no obvious indication of DNRA activity, or at least not at a level that exceeded the process of NH_4_^+^ assimilation. Therefore, the major NO_3_^−^- reducing pathway under the COD/NO_3_^−^-N ratio of 4/1 was assumed to be denitrification because depleted inorganic nitrogen was most likely converted to nitrogenous gases. The existence of DNRA in this system was subsequently verified with stable-isotope tracers (see below).

In the high C/NO_3_^−^ SBR, the COD/NO_3_^−^-N ratio was started at 8/1 and the system was observed in order to establish whether it has the ability to sustain the growth of DNRA microorganisms. After 4 d of operation, the NO_3_^−^ level was depleted (<0.2 mg N L^−1^). This rapid consumption of NO_3_^−^ was likely to be due to the higher glucose concentration in the high C/NO_3_^−^ reactor than in the low C/NO_3_^−^ reactor, which, in turn, required electron acceptors (NO_3_^−^ and NO_2_^−^) to complete the reactions catalyzed by the microorganisms. Therefore, NO_3_^−^ became a limiting substrate in this system, whereas NO_2_^−^ was undetectable throughout the experiment, suggesting the rapid conversion of NO_2_^−^ to other end products. The amount of NH_4_^+^ in the high C/NO_3_^−^ SBR was increased to a markedly higher level than the supplied concentration, possibly accounting for 30.1–64.4% and 68.6–100% of the NO_3_^−^ conversion during days 20–37 and 38–54, respectively. The active formation of NH_4_^+^ most likely indicates the occurrence of DNRA in this high C/NO_3_^−^ system. Judging from the portion of NO_3_^−^ converted to NH_4_^+^ during days 20–37, DNRA microorganisms and denitrifiers appeared to have a relatively similar share in NO_3_^−^ reduction. However, after day 37, DNRA microorganisms appeared to be the principal NO_3_^−^ reducers in the reactor, with a minor fraction of reduced NO_3_^−^ being released as gases (<14.5% during peak NH_4_^+^-forming activity). The diverged nitrogen profiles observed in the two SBRs indicated that the difference in the ratios selected for the low and high C/NO_3_^−^ reactors induced partitioning in the pathways of microbial NO_3_^−^ reduction. Since the COD/NO_3_^−^-N of 8/1 already supported the growth and activity of DNRA microorganisms, the ratio was not increased from this value. COD/NO_3_^−^-N of 7.7 was successfully used to enrich a DNRA community in an acetate-fed continuous culture (36). This range of COD/NO_3_^−^-N values appears to be sufficiently high to promote the reduction of NO_3_^−^ to NH_4_^+^. However, other parameters, such as the type of organic carbon and its fermentability, also need to be considered as additional factors that affect the COD/NO_3_^−^-N threshold partitioning the pathways of NO_3_^−^ reduction.

With respect to the cell biomass grown in the two SBRs, the MLSS in the high C/NO_3_^−^ system was greater (3,008±996 mg L^−1^) than that observed in the low C/NO_3_^−^ system (2,740±272 mg L^−1^). Since the same amount of NO_3_^−^ was supplied to both reactors, the higher cell mass in the high C/NO_3_^−^ system may be due to the higher mole of organic carbon required per mole of NO_3_^−^ for DNRA than for denitrification (32), thereby providing a greater carbon and energy source for microbial growth. It may also have been caused by the growth of other microorganisms apart from NO_3_^−^ reducers because an organic carbon concentration above the stoichiometric requirement for NO_3_^−^ reduction is expected to support other anaerobic heterotrophs, such as fermentative bacteria and SO_4_^2−^ reducers. Sulfide (S^2−^) produced from SO_4_^2−^ reduction may sustain DNRA microorganisms by acting as an electron donor (6). However, the effects of S^2−^ in the high C/NO_3_^−^ reactor may be small considering the amount of glucose added, with SO_4_^2−^ being supplied in the media at 24.6±0.66 mg S L^−1^. Therefore, if all the SO_4_^2−^ supplied was reduced to S^2−^, 1.35% (2.69±0.07 mg N L^−1^) of the added NO_3_^−^ would be used by S^2−^-driven DNRA (see Supplementary for details), and, hence, its effects on this process were expected to be negligible relative to those on the organic carbon-driven reaction.

### Potential activity of DNRA in sludge from low and high C/NO_3_^−^ SBRs

**Fig. 2:**
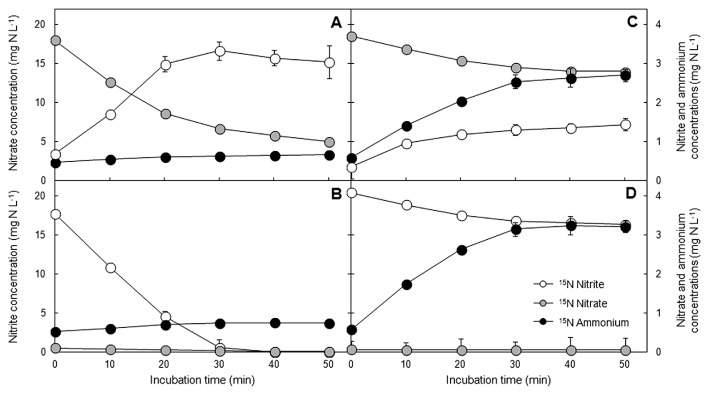
Changes in ^15^NO_3_^−^, ^15^NO_2_^−^, and ^15^NH_4_^+^ during stable-isotope tracer experiments; (A, C) ^15^NO_3_^−^ and (B, D) ^15^NO_2_^−^ incubations with (A, B) low and (C, D) high C/NO_3_^−^ sludge.

Batch incubations with low C/NO_3_^−^ sludge revealed that ^15^NO_3_^−^ and ^15^NO_2_^−^ were mostly reduced to end products that did not remain in the liquid phase (Fig. 2A, B), and, thus, they were assumed to be converted into nitrogenous gases. This was mostly attributed to the activity of denitrifiers, which produce nitric oxide (NO), nitrous oxide (N_2_O), and dinitrogen (N_2_) gases from the reduction of NO_3_^−^ and NO_2_^−^. However, a slight increase in ^15^NH_4_^+^ was measured with the ^15^NO_3_^−^ and ^15^NO_2_^−^ tracers, although at small concentrations only (0.19 and 0.23 mg N L^−1^, respectively). This weak activity indicated the presence of a small amount of DNRA microorganisms in the low C/NO_3_^−^ community. ^15^NH_4_^+^ and ^15^NO_3_^−^/^15^NO_2_^−^ assimilation in all the incubations was expected to be suppressed due to the presence of a high concentration of _14_NH_4_^+^ (100 mg N L^−1^). Therefore, this process was not taken into account for the balance of nitrogen conversion, and all of the ^15^NH_4_^+^ produced was assumed to be left in the media.

Regarding high C/NO_3_^−^ sludge, a marked increase in the level of ^15^NH_4_^+^ was observed when ^15^NO_3_^−^ or ^15^NO_2_^−^ was added (Fig. 2C, D). Therefore, DNRA activity was confirmed by the evidence of NH_4_^+^ production from the reduction of NO_3_^−^ and NO_2_^−^. The portion of consumed ^15^NO_3_^−^/^15^NO_2_^−^ not detected in the liquid phase was assumed to be converted to gaseous products. The addition of ^15^NO_2_^−^ instead of ^15^NO_3_^−^ did not significantly change the activity of DNRA microorganisms in the low and high C/NO_3_^−^ sludge incubations. Hence, NO_2_^−^ does not appear to have had a different effect on the nitrogen conversion pathways from that observed for NO_3_^−^, at least for the samples and conditions tested in this experiment.

**Fig. 3:**
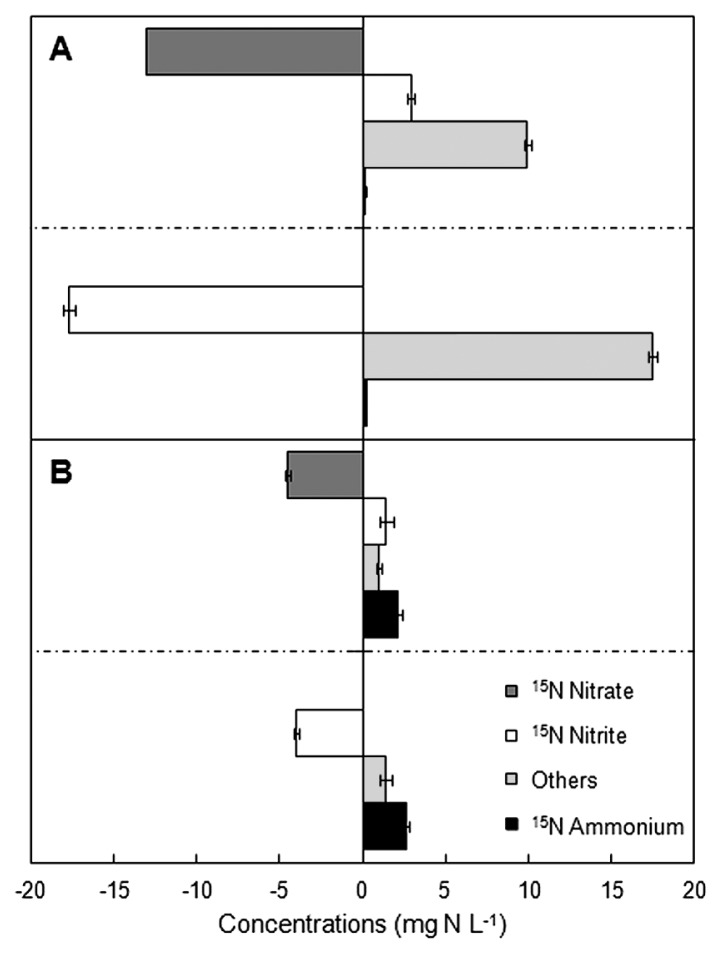
Net changes in ^15^N-nitrogen in ^15^NO_3_^−^ and ^15^NO_2_^−^ incubations with (A) low and (B) high C/NO_3_^−^ sludge. Data are shown with the standard deviation (error bar) derived from triplicate incubations of the sludge of each reactor.

Net changes in ^15^N-nitrogen for all incubations (calculated based on the initial and final concentrations of ^15^NO_3_^−^, ^15^NO_2_^−^, and ^15^NH_4_^+^) are shown in Fig. 3. The main products of the reduction of ^15^NO_3_^−^ and ^15^NO_2_^−^ in low C/NO_3_^−^ sludge were in gaseous forms (Fig. 3A), and, thus, denitrifiers were assumed to be the main contributors to nitrogen conversion in these samples. The ^15^NH_4_^+^ produced from DNRA microorganisms inhabiting the low C/NO_3_^−^ SBR was 1.4 and 1.3% of the amount of ^15^NO_3_^−^ and ^15^NO_2_^−^ consumed, respectively, indicating a small role for these microorganisms in the denitrifier-dominated community. In contrast, the main product in high C/NO_3_^−^ sludge was ^15^NH_4_^+^ in the ^15^NO_3_^−^ and ^15^NO_2_^−^ incubations (Fig. 3B). The proportions of nitrogenous gases produced in the high C/NO_3_^−^ incubations (21.9 and 34.5% of consumed ^15^NO_3_^−^ and ^15^NO_2_^−^, respectively) were lower than those converted to ^15^NH_4_^+^. However, these gases may not come exclusively from the activity of denitrification. Previous studies revealed that DNRA also released NO and N_2_O (30, 39, 42); however, the exact production pathways remain unclear. Since high C/NO_3_^−^ sludge was predominated by DNRA activity, the nitrogenous gases produced may come from denitrification, DNRA, or both.

At the end of the incubations, the net ^15^NH_4_^+^ generated in the high C/NO_3_^−^ sludge was 46.6 and 65.5% of the consumed ^15^NO_3_^−^ and ^15^NO_2_^−^, respectively. The extent of ^15^NH_4_^+^ formed here suggested the competitive potential of DNRA microorganisms in activated sludge when under a continuous supply of high C/NO_3_^−^ loading. Additionally, NO_3_^−^ and NO_2_^−^ had the ability to induce DNRA in a similar manner; however, these two nitrogen species were previously reported to exert different stimulating effects on the pathway in *Escherichia coli* (26, 41). In conclusion, the results from stable isotope tracers confirmed that NH_4_^+^ detected in the high C/NO_3_^−^ SBR was mainly the product of DNRA, and that the addition of glucose at a COD/NO_3_^−^-N ratio of 8/1 may be selected for the growth and activity of DNRA microorganisms; however, denitrifiers may still co-exist to a certain level.

### Microbial communities of low and high C/NO_3_^−^ SBRs

**Fig. 4:**
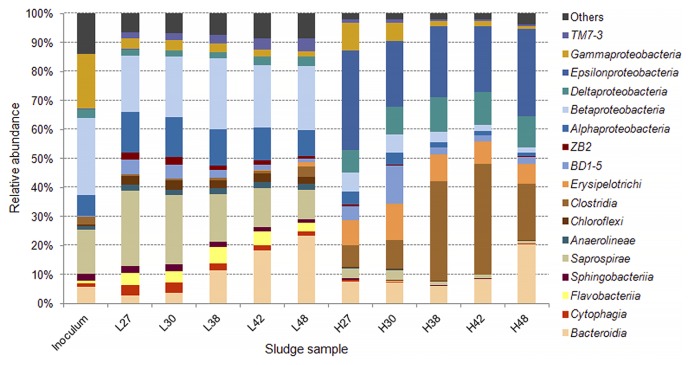
Relative abundance of dominant classes in the inoculum and low C/NO_3_^−^ (L27, L30, L38, L42, and L48) and high C/NO_3_^−^ (H27, H30, H38, H42, and H48) sludge samples.

**Fig. 5:**
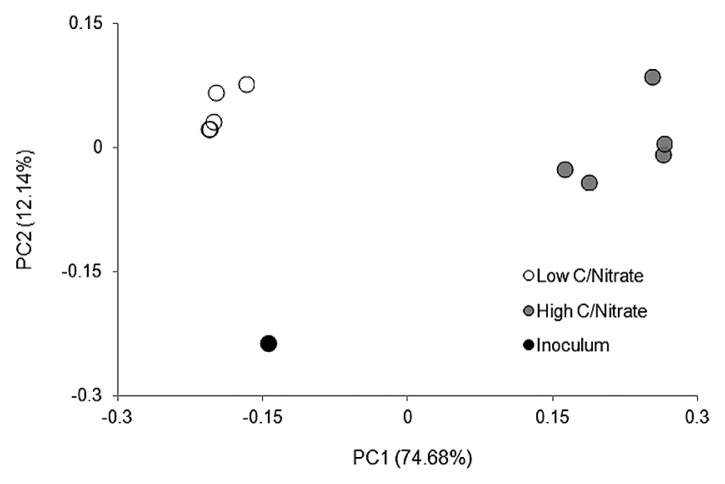
PCoA plot of beta-diversity measures of the inoculum and low and high C/NO_3_^−^ microbial communities.

**Fig. 6:**
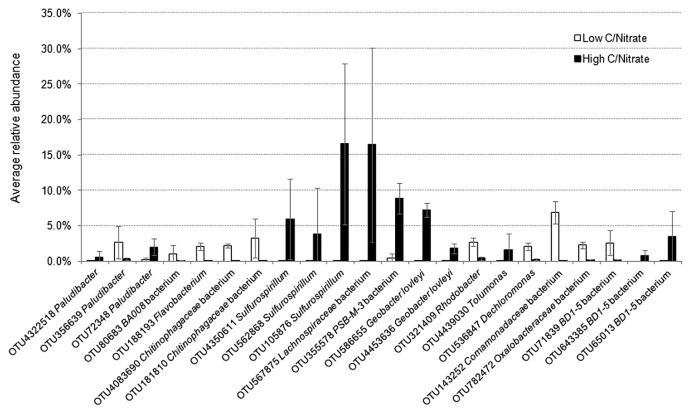
Average relative abundance of top 22 OTUs identified in low and high C/NO_3_^−^ SBRs (average from five sampling days). Data are shown with the standard deviation (error bar) among the sampling days of each sludge sample.

An Illumina MiSeq 16S rRNA analysis revealed that from all the samples collected, 98.4–99.8% of sequences were identified as *Bacteria* and ≤0.01% as *Archaea*, while it was not possible to designate 0.2–1.6% of the reads. The OTUs assigned from the sequence reads were classified into 17 main classes (Fig. 4) belonging to seven phyla, including *Proteobacteria*, *Firmicutes*, *Bacteroidetes*, *Chloroflexi*, and the candidate divisions GN02, OD1, and TM7. Microbial richness, as estimated by the Chao1 index, and microbial diversity, as calculated by the Shannon index, revealed a significant difference (*P*<0.05) between the low and high C/NO_3_^−^ microbial populations (see Table S2). Lower richness and biodiversity were observed in high C/NO_3_^−^ samples, whereas low C/NO_3_^−^ populations were more diverse with higher community richness. Therefore, a higher C/NO_3_^−^ ratio appeared to allow certain bacteria to thrive with elevated proportions, while the lower C/NO_3_^−^ ratio nurtured diverse bacteria that, among the dominating ones, had relatively fair shares of the microbial composition, as indicated by the relative abundance of the major OTUs in the system (see Fig. S3). The relationships between the inoculum and the low and high C/NO_3_^−^ microbial communities were revealed in the PCoA plot (Fig. 5) using a beta-diversity measure of each sample. The PCoA plot showed distinct clustering within the low and high C/NO_3_^−^ samples, with the distance between the beta-diversities of the two reactors’ populations conveying the differences in microbial compositions among these two ecosystems. The average relative abundance of the major taxa identified (Fig. 6) indicated that the microbial communities of the low and high C/NO_3_^−^ SBRs were distinct from each other with rarely shared OTUs among them, which reflects partitioning in the NO_3_^−^-reducing activities observed in the two systems.

Among the top OTUs identified in the high C/NO_3_^−^ reactor, those with the potential of being DNRA microorganisms included those closely related to the genus *Sulfurospirillum*, the family *Lachnospiraceae*, the proposed genus PSB-M-3, and the species *Geobacter lovleyi*. These taxa were present at an average abundance of ≤0.4% (from the five sampling days) in the low C/NO_3_^−^ system, which emphasized their roles in shaping the NH_4_^+^-producing community in the high C/NO_3_^−^ SBR. The OTU affiliated with *Sulfurospirillum* (OTU105876) was one of the OTUs detected with the highest average abundance in the high C/NO_3_^−^ reactor (16.5%). The genus *Sulfurospirillum* (belonging to the class *Epsilonproteobacteria*) is known to exhibit versatile metabolism ranging from SO_4_^2−^ reduction, S^2−^ oxidation, and DNRA (18, 25, 31). However, the amounts of NO_3_^−^ and organic carbon available in the high C/NO_3_^−^ SBR were expected to promote NO_3_^−^ reduction to NH_4_^+^ in these microorganisms, while the influence of sulfur compounds was expected to be minimal because the level of sulfur-driven NO_3_^−^ reduction in the reactor was small. Three OTUs related to *Sulfurospirillum* were found at a high abundance in the high C/NO_3_^−^ system, but were present at ≤0.02% of the total community in the low C/NO_3_^−^ reactor, which indicated that the high C/NO_3_^−^ condition was highly selective for the growth of these populations. The family *Lachnospiraceae* (in the class *Clostridia* of the phylum *Firmicutes*), the other highest average abundant OTU (16.5%) (OTU567875), is not known to contain DNRA microorganisms, but was previously found in anoxic microcosms performing DNRA (23). Apart from the highest OTU, another top OTU in the high C/NO_3_^−^ SBR was related to PSB-M-3 (OTU355578) in the family *Erysipelotrichaceae* of the phylum *Firmicutes*, with an average abundance of 8.9%. The high proportions of the taxa affiliated with *Lachnospiraceae* and PSB-M-3 suggest their essential role in the high C/NO_3_^−^ ecosystem; however, further investigations are needed in order to elucidate their actual ecological function in NO_3_^−^-reducing communities.

The OTU assigned to the species *G. lovleyi* (OTU586655), a member of the class *Deltaproteobacteria*, was detected with an average abundance of 7.2% in the high C/NO_3_^−^ reactor. *G. lovleyi* is known to conduct DNRA (33) and taxa related to this species have also been found to dominate DNRA enrichment cultures fed with acetate (36–38). Previous findings and the present results indicate the common occurrence of these DNRA microorganisms when the conditions favor NO_3_^−^ reduction to NH_4_^+^. Beside those with DNRA potential, the remaining major OTUs were found to be related to bacteria with fermentative abilities, including the OTUs assigned to the class BD1-5 (OTU65013), genus *Tolumonas* (OTU4439030), and genus *Paludibacter* (OTU72348). There is no direct evidence to show that these taxa perform NO_3_^−^ reduction (9, 16, 35), and, thus, they most likely acted as contributors supplying simpler fermented products to the rest of the community.

In the low C/NO_3_^−^ SBR, the OTU detected with the highest average abundance (6.9%) was assigned to the family *Comamonadaceae* (OTU143252) in the class *Betaproteobacteria*. Several members of this family have been recognized as denitrifying bacteria, such as *Comamonas* and *Acidovorax* (43). Other OTUs affiliated with known denitrifiers include those related to *Rhodobacter* (OTU321409), *Dechloromonas* (OTU536847), and *Flavobacterium* (OTU188193) (5, 8, 11). OTUs related to the families *Chitinophagaceae* (OTU181810 and OTU4083690) and *Oxalobacteraceae* (OTU782472) were also identified in the low C/NO_3_^−^ community, in which some members are known to reduce NO_3_^−^ or perform complete denitrification (20). The detection of these major OTUs in the low C/NO_3_^−^ reactor was in accordance with the denitrifying activity observed in the system. Additionally, certain denitrifiers, such as *Shewanella loihica* and several *nirK*-containing bacteria, have been found to contain the DNRA pathway in their genomes (17, 44). Therefore, the weak activity of DNRA detected in the low C/NO_3_^−^ SBR may be attributed to one of these denitrifiers because no other known DNRA microorganism was identified. Between the two NO_3_^−^-reducing ecosystems, smaller changes in the microbial community were observed for the low C/NO_3_^−^ reactor (in which the enriched populations were still denitrifiers), while the high C/NO_3_^−^ condition was enriched for a markedly different microbial composition from the inoculum (see Fig. S4). This result demonstrated that the selected C/NO_3_^−^ ratios applied to the two SBRs were already critical for the partition of microbial populations to those with different functional abilities.

## Conclusion

In the high C/NO_3_^−^ SBR, DNRA was observed by the marked increase in the NH_4_^+^ concentration. Nitrogen conversion, monitored via ^15^NO_3_^−^ and ^15^NO_2_^−^ tracers, revealed that DNRA microorganisms were the major contributors to NO_3_^−^/NO_2_^−^ reduction under a high COD/NO_3_^−^-N ratio of 8/1, at which NO_3_^−^ and NO_2_^−^ induce DNRA in a similar manner. With a low COD/NO_3_^−^-N ratio of 4/1, denitrifiers were the major NO_3_^−^/NO_2_^−^ reducers. Additionally, the high C/NO_3_^−^ conditions enriched for microbial populations markedly differed from those in the low C/NO_3_^−^ reactor and the inoculum. These populations were comprised of OTUs closely related to known DNRA microorganisms (*Sulfurospirillum* and *G. lovleyi*) as well as fermentative bacteria and those that may be capable of both functions. These bacteria were rarely present in the low C/NO_3_^−^ system, which harbored a community of denitrifiers. Therefore, the C/NO_3_^−^ ratios applied to each SBR provided an environment that partitioned the pathways of NO_3_^−^ reduction to either nitrogenous gases or NH_4_^+^ as the end product, which was the result of the distinctive microbial composition found in each SBR system.

## Supplementary Material
