# Supplementary material for: Distinct Microbial Community Performing Dissimilatory Nitrate Reduction to Ammonium (DNRA) in a High C/NO3− Reactor
Source: Microbes Environ. 2018 Sep 29;33(3):264–71. doi: 10.1264/jsme2.ME17193 (PMC6167113; doi:10.1264/jsme2.ME17193)
Supplement: Supplementary file 1 [file 33_264_s1.pdf]

Table S1. Glucose and NO_3^- supplied to the low and high C/NO_3^- SBRs during the 6-h and 12-h cycle periods

SBRs	COD/NO_3^--N ratio	During 6-h SBR cycle		During 12-h SBR cycle	
		Glucose (mg L^{-1})	NO_3^- (mg-N L^{-1})	Glucose (mg L^{-1})	NO_3^- (mg-N L^{-1})
Low C/NO_3^-	4/1	400	100	800	200
High C/NO_3^-	8/1	800	100	1,600	200

Table S2. Microbial community richness (Chao1 index) and biodiversity (Shannon index) of the samples taken from the inoculum and the low and high C/NO_3^- SBRs

Index	Inoculum	L27	L30	L38	L42	L48	H27	H30	H38	H42	H48
Chao1	3688.6	4288.4	4720.5	4772.0	4978.1	5024.6	3872.5	3533.4	2855.1	2401.4	3157.5
Shannon	8.10	7.84	7.78	7.86	7.98	7.90	5.30	5.48	4.57	4.48	5.70

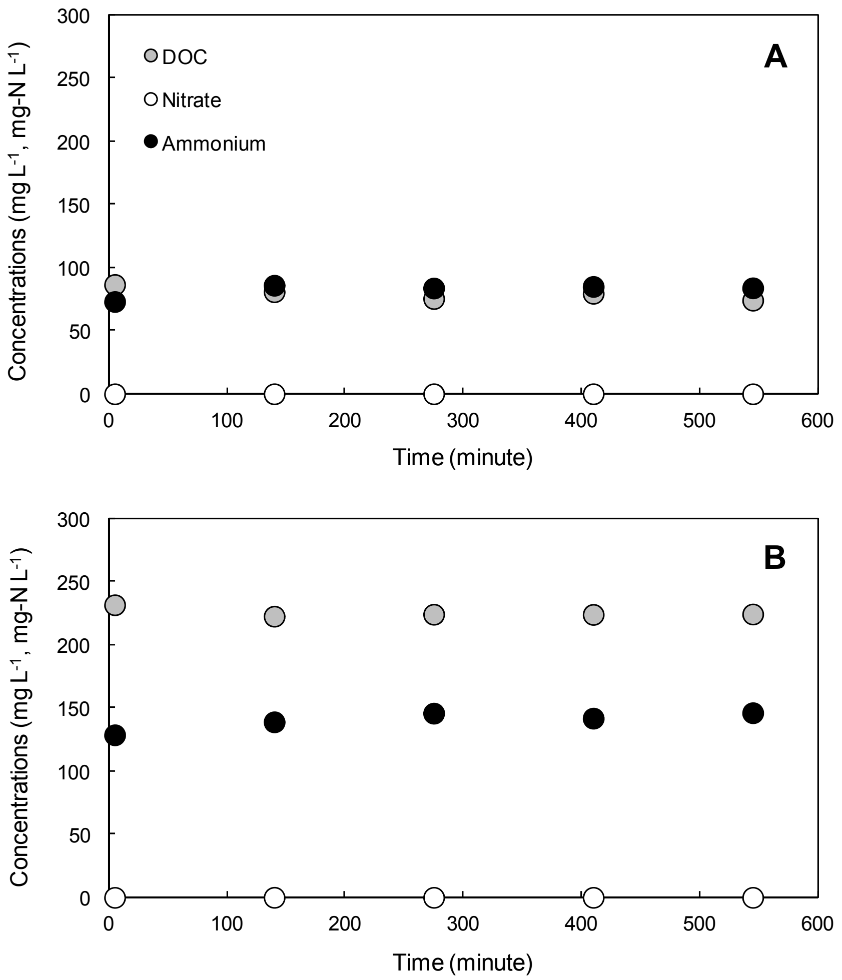

Fig. S1. Stable NO_3^-, NH_4^+ and DOC concentrations observed during one cycle of the (A) low and (B) high C/NO_3^- SBRs (on day 20)

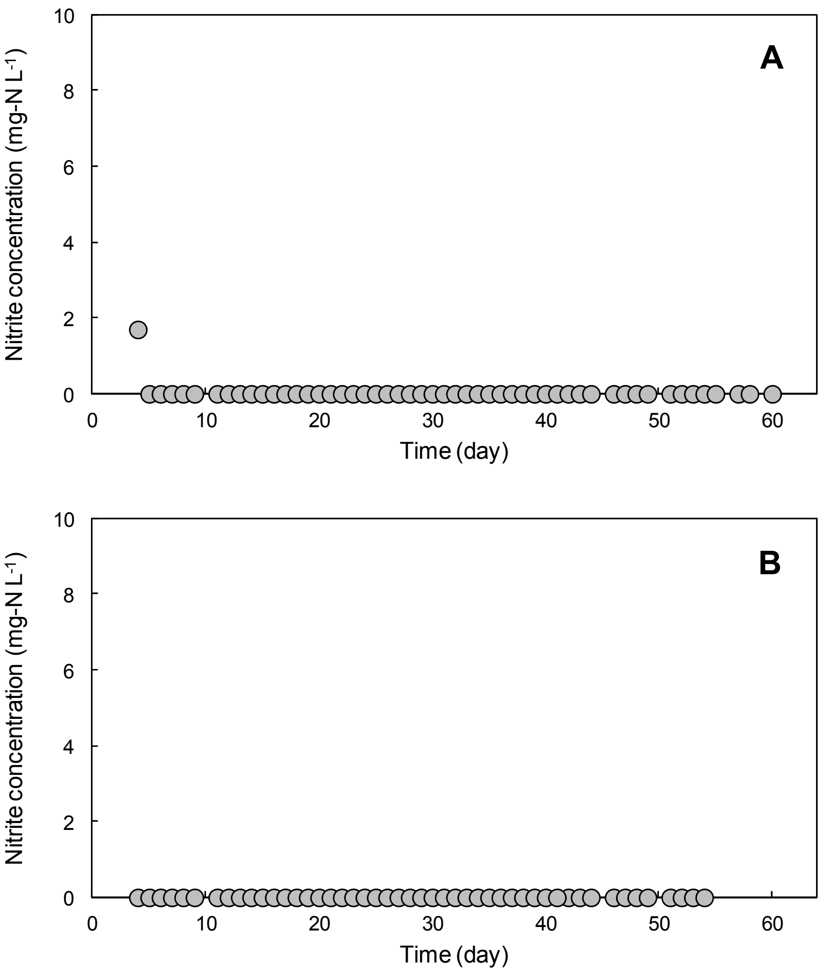

Fig. S2. NO_2^- concentrations measured in the (A) low and (B) high C/NO_3^- SBRs

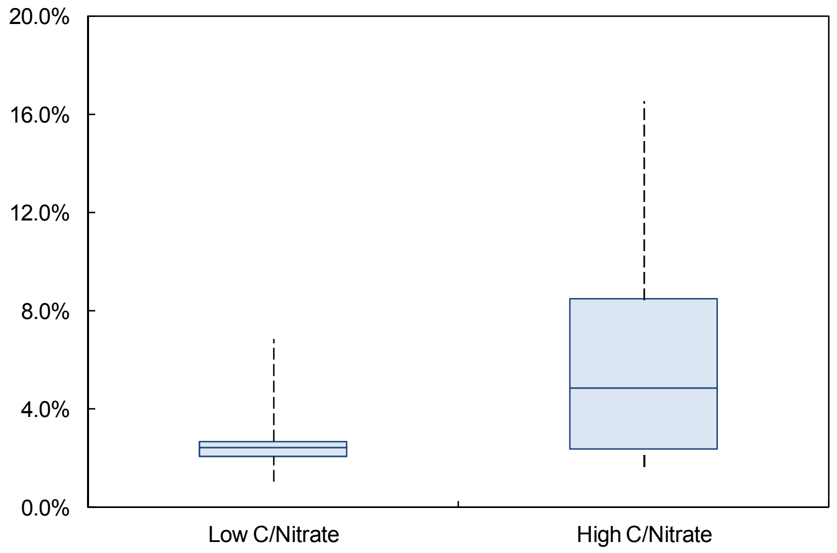

Fig. S3. Box plot depicting variation in relative abundances of the major OTUs in the low and high C/NO_3^- sludge samples

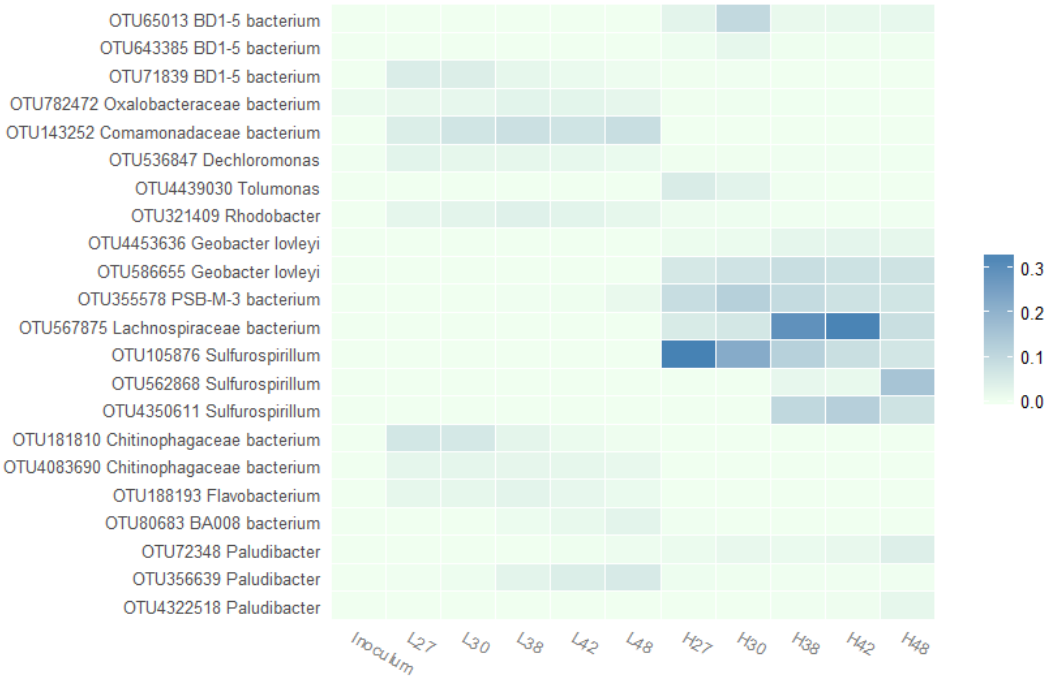

Fig. S4. Heatmap showing relative abundances of the top 22 OTUs in the inoculum, the low C/NO_3^- (L27, L30, L38, L42 and L48) and the high C/NO_3^- (H27, H30, H38, H42 and H48) SBRs (the scale is in a range 0.0 to 1.0)

Calculation on the sulfide-driven DNRA

DNRA pathway with sulfide as an electron donor can be described as follows,

$$4\ HS^- + NO_3^- + 6\ H^+ \rightarrow NH_4^+ + 4\ S^0 + 3\ H_2O \qquad (1)$$

The DNRA reaction requires four moles of sulfide per one mole of NO_3^-. Therefore, if all the supplied SO_4^{2-} (24.6±0.66 mg-S^{2-} L^{-1}) was to be reduced to sulfide, 2.69±0.07 mg-N L^{-1} of NO_3^- would be used by the sulfide-driven DNRA reaction, which is equal to 1.35% of the NO_3^- added (200 mg-N L^{-1}). The DNRA pathway that could be induced by sulfide hence should be negligible considering the portion of NO_3^- that could be utilized by the process.
